# Supplementary material for: Heparan sulfate selectively inhibits the collagenase activity of cathepsin K
Source: bioRxiv. 2024 Jan 8:2024.01.05.574350. Preprint. [Version 2] doi: 10.1101/2024.01.05.574350 (PMC10802503; doi:10.1101/2024.01.05.574350)
Supplement: Supplement 1 [file media-1.pdf]

Figure S1

A

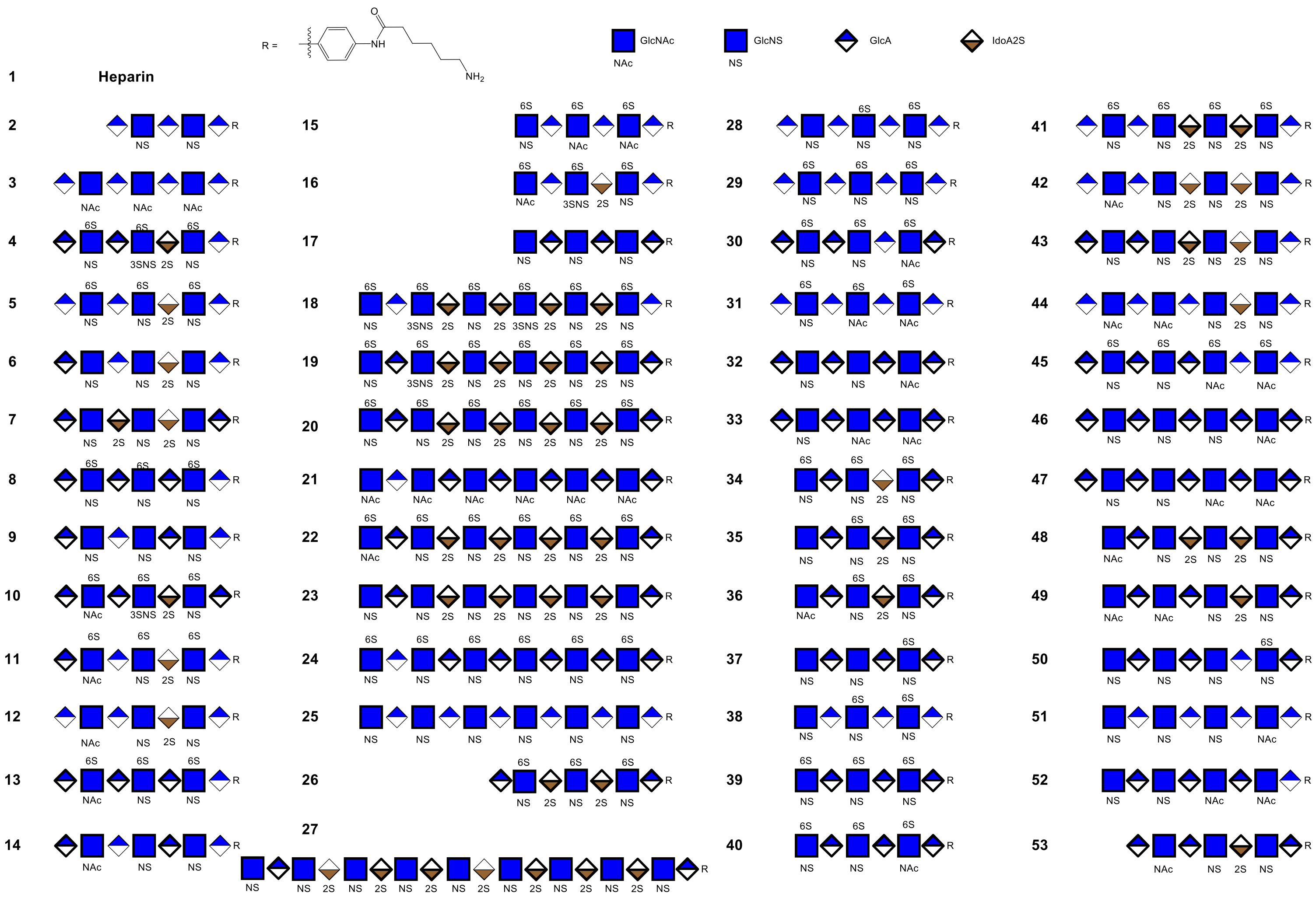

Figure S1. Structures of HS oligosaccharides immobilized on the microarray.

Figure S2

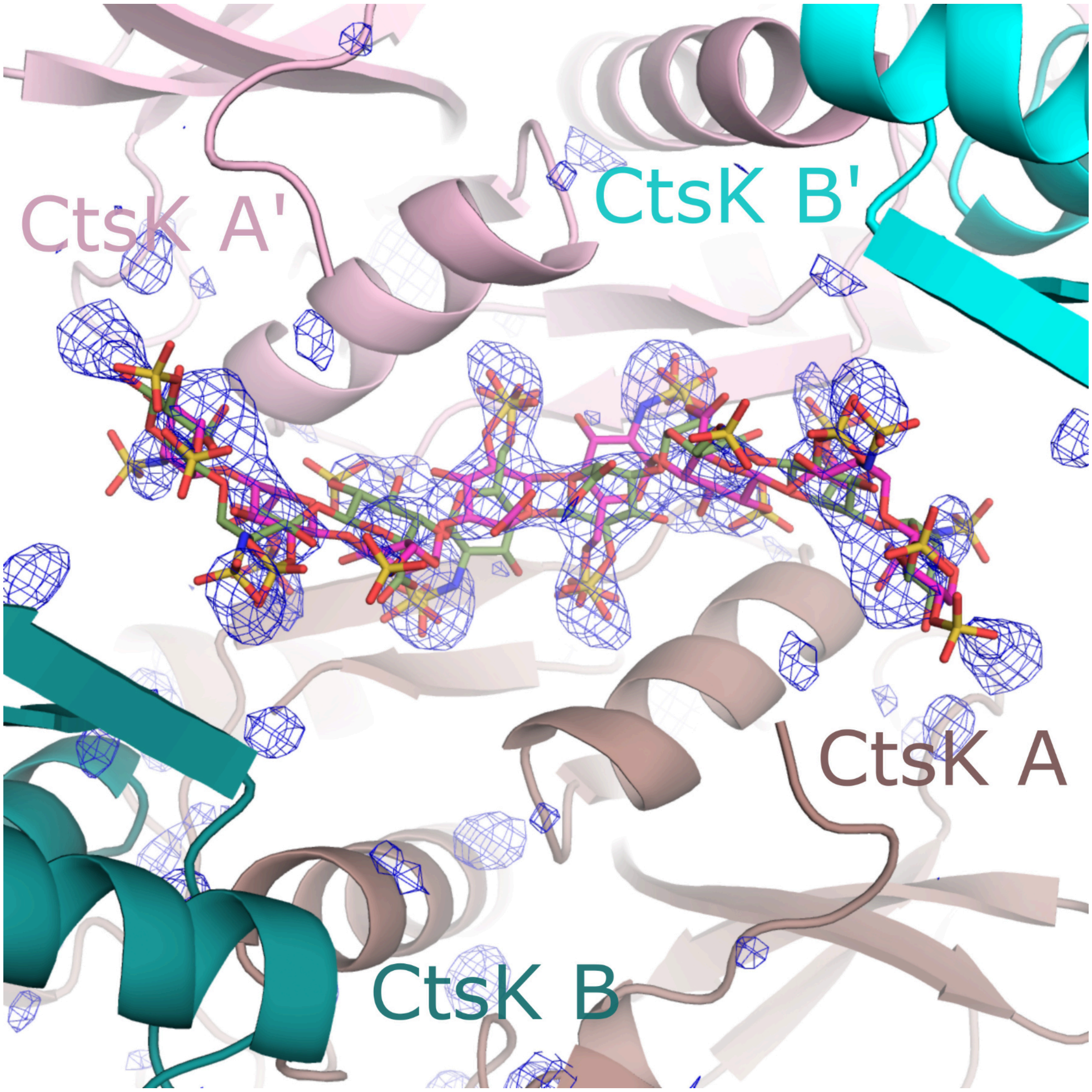

Figure S2. Electron density of Heparan sulfate bound to CtsK. Simulated annealing fo-fc omit density for the heparan sulfate oligomer bound in the Ctsk-12merNS2S6S structure is displayed contoured at 3σ. Symmetry related HS 12mers are shown in magenta and light green, respectively.

Figure S3

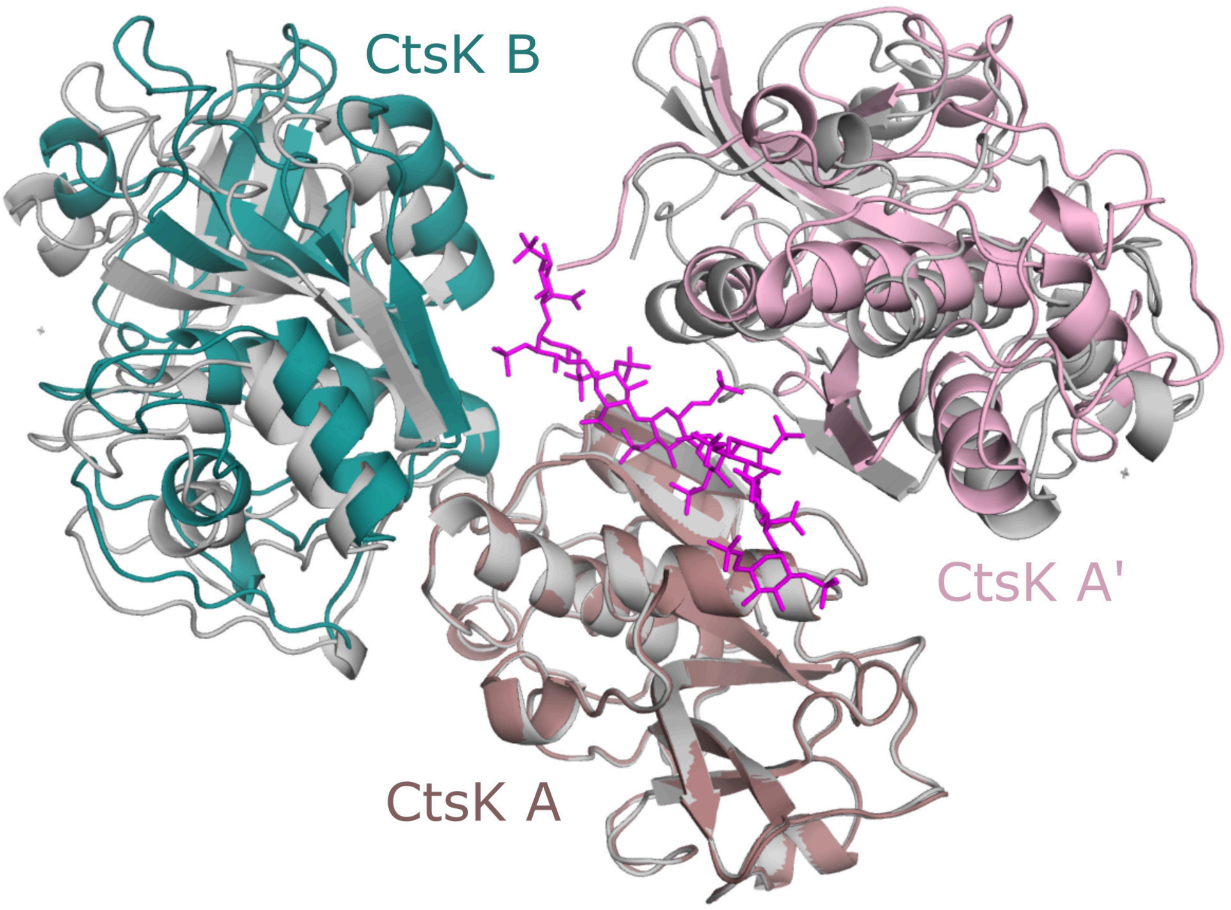

Figure S3. Similar interfaces in the CtsK+12merNS2S6S and the CtsK+Cst-3+12merNS2S3S6S structures. Superposition of monomer A from the crystallographic tetramer of Ctsk+12merNS2S6S (all molecules in grey, oligosaccharide in magenta) onto monomer A of the crystallographic tetramer found in the CtsK+Cst-3+12merNS2S3S6S present structure (monomers colored as in Figure 5). The symmetry related B' molecules from both structures and the Cst-3s have been removed for easy of viewing. Similar crystallographic interfaces exist in both structures.

Figure S4

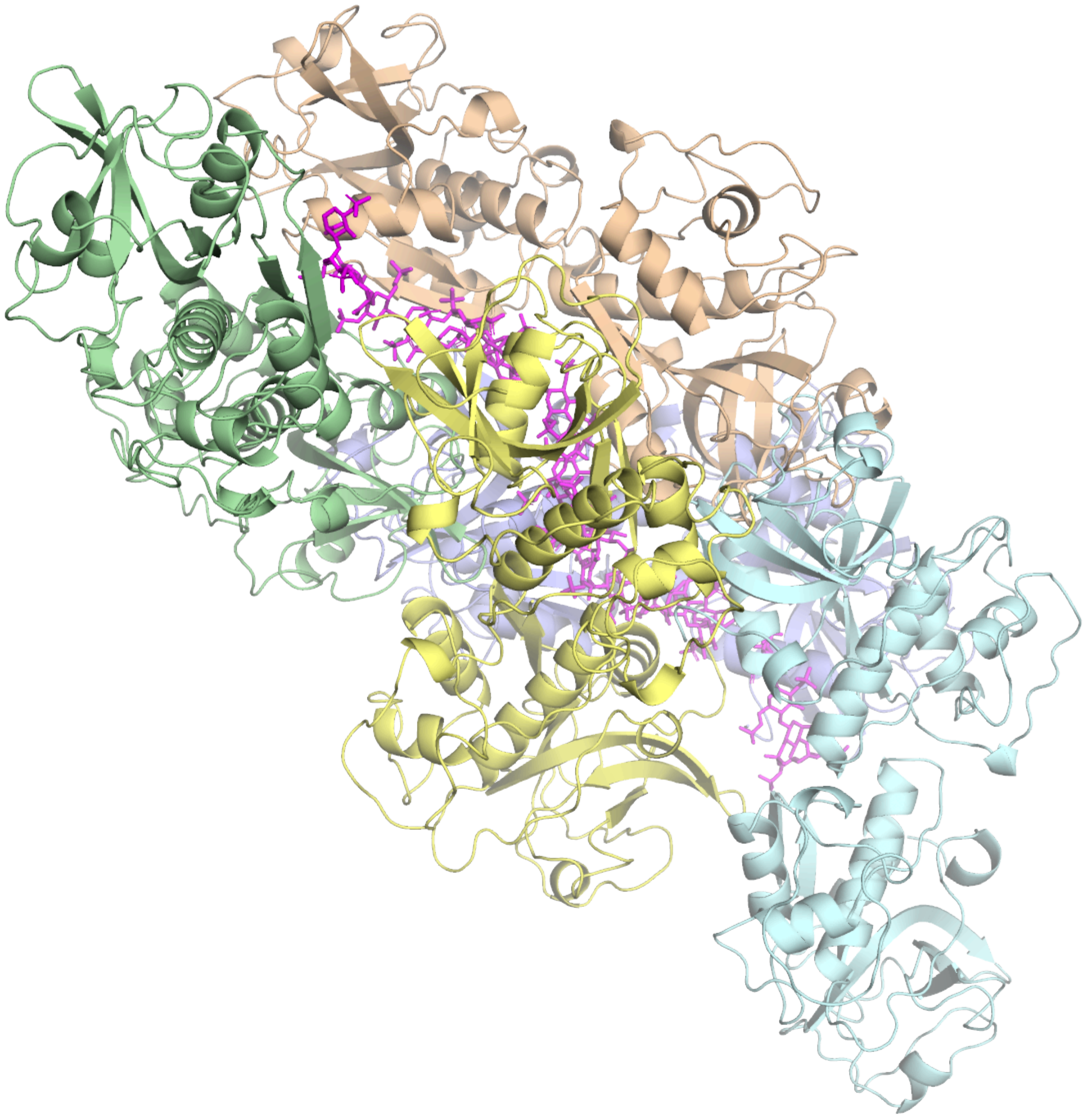

Figure S4. Theoretical helical packing of CtsK along HS. A theoretical model of CtsK binding to HS can be generated without much overlap by repeating the AB dimers (shown in pale cyan, light blue, light yellow, wheat and light green) with the same positioning as found at the crystallographic interface of the AA' dimer. The heparan is positioned based on the superposition of the CtsK. This model would allow for efficient packing of CtsK along the heparan sulfate chain.

Table S1. Crystallographic data and refinement statistics

	CstK + 12merNS2S6S	CstK + CycC + HS[a]
PDB ID code	8V58	8V57
**Data collection**	201116_SB3PN9	201009_SB1PN12
Space group	$P4_122$	$P222_1$
Cell dimensions		
a, *b*, *c* (Å)	111.892, 111.982, 116.653	92.93, 179.61, 73.325
α, β, γ (°)	90, 90, 90	90, 90, 90
Resolution (Å)	50/0-3.1 (3.15-3.1)[b]	50.00-2.75 (2.80-2.75)
R_{sym} (%)	9.2 (96.3)	5.9 (77.0)
I / σI	5.4 (1.67)	7.1 (1.66)
Completeness (%)	98.8 (99.6)	92.6 (86.6)
Redundancy	7.4	7.3 (7.1)
**Refinement**		
Resolution (Å)	19.87-3.10	38.35-2.75
No. reflections	13,647	30,119
R_{work} / R_{free} (%)	23.59/29.26	22.86/25.79
No. atoms		
Protein	3,169	9,813
Water	1	13
Other	252	115
B-factors		
Protein	113.75	83.15
Water	99.06	69.18
Other	113.46	95.71
R.m.s. deviations		
Bond lengths (Å)	0.009	0.007
Bond angles (°)	1.298	0.723
Ramachandran Plot[c]		
Allowed (%)	4.93	5.22
Favored (%)	95.07	94.32

a) 12merNS2S3S6S was included in the crystallization but is not modeled in the structure due to the density being ambiguous.
b) High resolution shell is shown in parentheses.
